# *LINC01088* inhibits tumorigenesis of ovarian epithelial cells by targeting *miR-24-1-*5*p*

**DOI:** 10.1038/s41598-018-21164-9

**Published:** 2018-02-13

**Authors:** Weijiang Zhang, Jing Fei, Shuqian Yu, Jiayu Shen, Xiaoqing Zhu, Annapurna Sadhukhan, Weiguo Lu, Jianwei Zhou

**Affiliations:** 1 Department of Gynecology, the Second Affiliated Hospital, College of Medicine, Zhejiang University, Hangzhou, Zhejiang 310051 China; 2 Department of Gynecologic Oncology, Women’s Hospital, College of Medicine, Zhejiang University, Hangzhou, Zhejiang 310006 China

## Abstract

The roles of long non-coding RNAs (lncRNAs), a class of long non-protein-coding RNAs, in the tumorigenesis of ovarian epithelial cells remain unknown. In this study, we discovered that the expression of *long intergenic non-coding RNA 1088* (*LINC01088)* was clearly reduced in benign epithelial ovarian tumor tissues compared to matched normal ovarian tissues. This was shown by global cDNA gene chip scanning and real-time qPCR, and validated in 42 clinical specimens. Furthermore, we found that *LINC01088* inhibited the growth of ovarian cancer xenografts in nude mice. Correlation analysis between *LINC01088* and mircoRNAs (miRNAs) conducted using primary clinical samples and RNA co-precipitation experiments revealed that *miR-24-1-*5*p* was one of the targets of *LINC01088*. Overexpression of *miR-24-1-*5*p* facilitated cell proliferation both *in vitro* and *in vivo*, however, *LINC01088* could partially reverse the cell proliferation induced by *miR-24-1-*5*p*. Finally, we demonstrated that *p21 activated kinase 4 (PAK4)* was one of the downstream key targets of *miR-24-1-*5*p* by luciferase reporter assay and Western blotting; and our results showed a remarkable decrease in cell proliferation after overexpression of PAK4. We conclude that *LINC01088* might function as a tumor suppressor, inhibiting the tumorigenesis of ovarian epithelial cells through *LINC01088*/ *miR-24-1-5p*/ *PAK4* axis.

## Introduction

Ovarian neoplasm is one of the most common gynecological tumors. The benign ovarian tumors can be cured by excision. Epithelial ovarian cancer is among the most lethal gynecological malignancies, with the five-year survival rate of less than 30%^1^. Despite constant improvement in surgical techniques and chemotherapy regimens for epithelial ovarian cancers in the past few years, the survival rates have not improved significantly^1^. Thus, it is essential to decipher the mechanism of ovarian epithelial tumorigenesis, in order to suppress tumor progression efficiently.

Long non-coding RNAs (LncRNAs) and mircoRNAs (miRNAs) are two classes of common non-protein-coding RNAs. LncRNAs are transcripts longer than 200 nucleotides^2^ that are involved in various biological processes, such as transcription regulation, cell proliferation and differentiation^3^. Also, lncRNAs play a crucial role in cancer development and progression^4–7^. MiRNAs are composed of approximately 18 to 22 nucleotides, and they play a role in biological processes like cell proliferation, apoptosis and cell differentiation^8,9^. It has been confirmed that aberrant expression of miRNAs is not only closely related to tumor progression, but also to tumorigenesis and neoplasm metastasis^10,11^. Studies have shown that lncRNAs contain miRNA response elements (MRE), which can bind to their target miRNAs to inhibit their biological functions (molecule “sponges”)^12^. Amanda *et al*.^13^ confirmed that lncRNA-*H19* bound to endogenous *let-7* by acting as a molecular sponge, which de-repressed the effect of endogenous *let-7* on targeting *high mobility group A2* (*Hmga2)* and promoted embryonic carcinoma cell proliferation and invasion. To identify the genes involved in tumorigenesis of ovarian epithelial cells, we conducted the global cDNA gene chip scanning and found that the expression of *long intergenic non-coding RNA 1088* (*LINC01088)* in benign epithelial ovarian tumor tissues was reduced in comparison to normal ovarian epithelial tissues. Further exploration indicated that *miR-24-1-5p* was a target of *LINC01088*, which provided experimental support for the role of *LINC01088* in effective suppression of the occurrence of epithelial ovarian cancers.

## Results

### *LINC01088* is down-regulated in benign epithelial ovarian tumors

**Figure 1:**
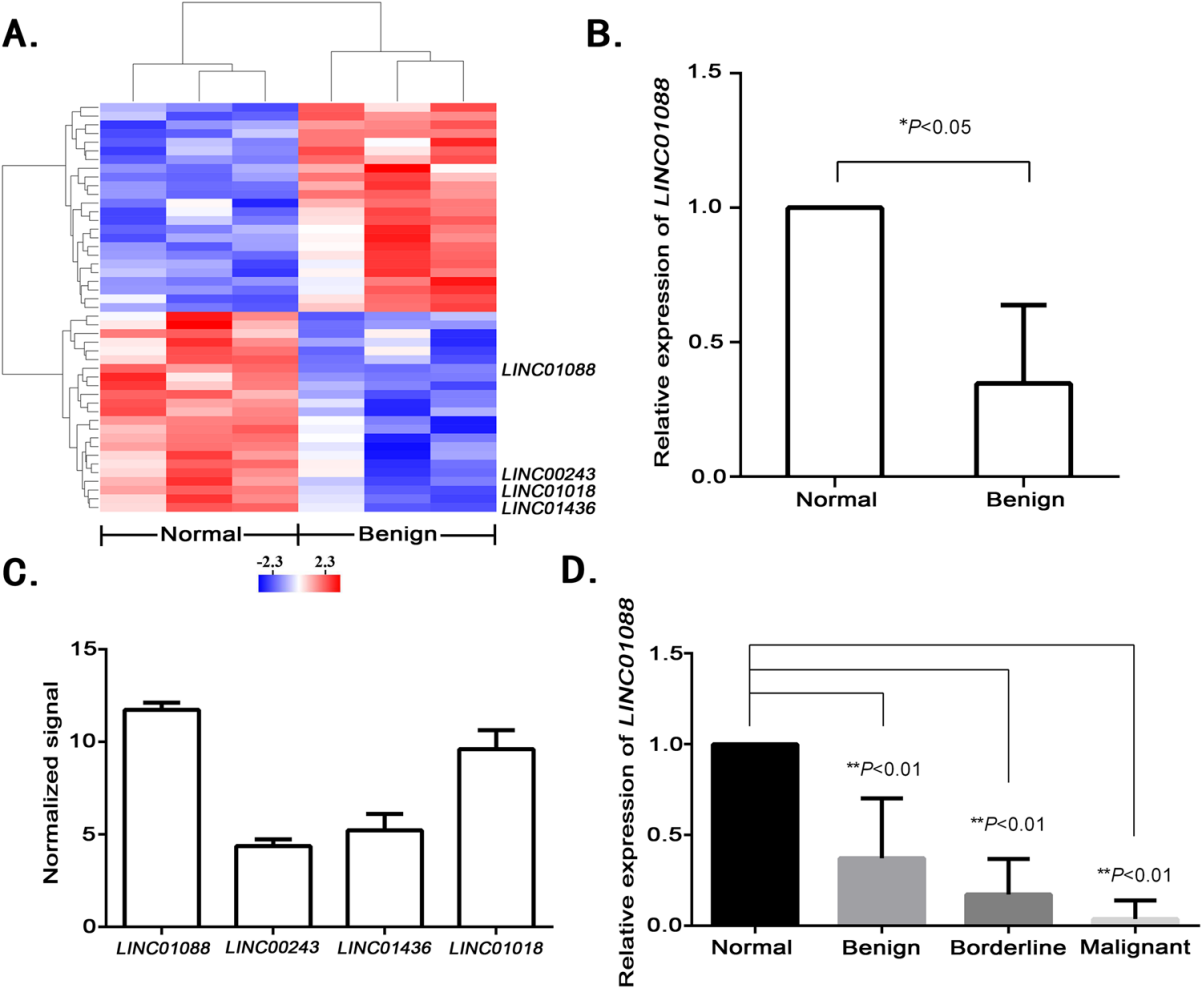
*LINC01088* is down-regulated in benign epithelial ovarian tumors. (**A**) LincRNA screening involved in tumorigensis. LincRNA gene chip was performed in 3 benign epithelial ovarian tumor tissue specimens and the matched normal tissues. (**B**) Validation of *LINC01088*. RNA was isolated from the clinical specimens using Trizol. After reverse transcription, real-time qPCR was conducted to determine the *LINC01088* level in 3 benign epithelial ovarian tumor tissue specimens and the matched normal tissues. Data are represented as mean +/− SD, *means *P* < 0.05 *vs* normal group (*ANOVA*). (**C**) The expressive abundance of four lincRNAs. Normalized signals of *LINC01088*, *LINC00243*, *LINC01436* and *LINC01018* in normal ovarian epithelial tissues showed by gene chip above. (**D**) *LINC01088* in primary epithelial ovarian tumors. RNA was isolated from the clinical specimens using Trizol. After reverse transcription, real-time qPCR was conducted to determine the *LINC01088* level in 12 benign epithelial ovarian tumor tissue specimens, 8 borderline epithelial ovarian tumor tissue specimens, 12 malignant epithelial ovarian tumor tissue specimens and the matched normal tissues. Data are represented as mean +/− SD, **means *P* < 0.01 vs normal group (*ANOVA*).

To evaluate the main lincRNAs involved in tumorigenesis of ovarian epithelial cells, we analysed lincRNA expression in 3 benign epithelial ovarian tumor tissue specimens and 3 normal ovarian epithelial tissue specimens using gene chip scanning. Compared with the normal group, the expression levels of the most significant four lincRNAs (*LINC01088*, *LINC01018*, *LINC01436* and *LINC00243*) were remarkably down-regulated in the tumor group (Fig. 1A), and the down-regulation of *LINC01088* was the most marked. Also, RT-qPCR on the same tissue specimens gave similar results with the gene chip analyses (Fig. 1B). Next, we compared the expression levels of these four lincRNAs in normal ovarian epithelial tissue specimens, and found that the expression level of *LINC01088* was the highest (Fig. 1C). Thus, we chose *LINC01088* for further study. Moreover, in an experiment with increased sample size, we discovered once again that the expression of *LINC01088* was prominently decreased in benign epithelial ovarian tumor tissues compared to the normal tissues. Additionally, *LINC01088* expression declined even more in borderline and malignant epithelial ovarian tumors (Fig. 1D). The above results suggested that *LINC01088* was a molecule that was negatively associated with tumorigenesis and might be a tumor suppressor gene.

### *miR-24-1-5p* is a target of *LINC01088*

**Figure 2:**
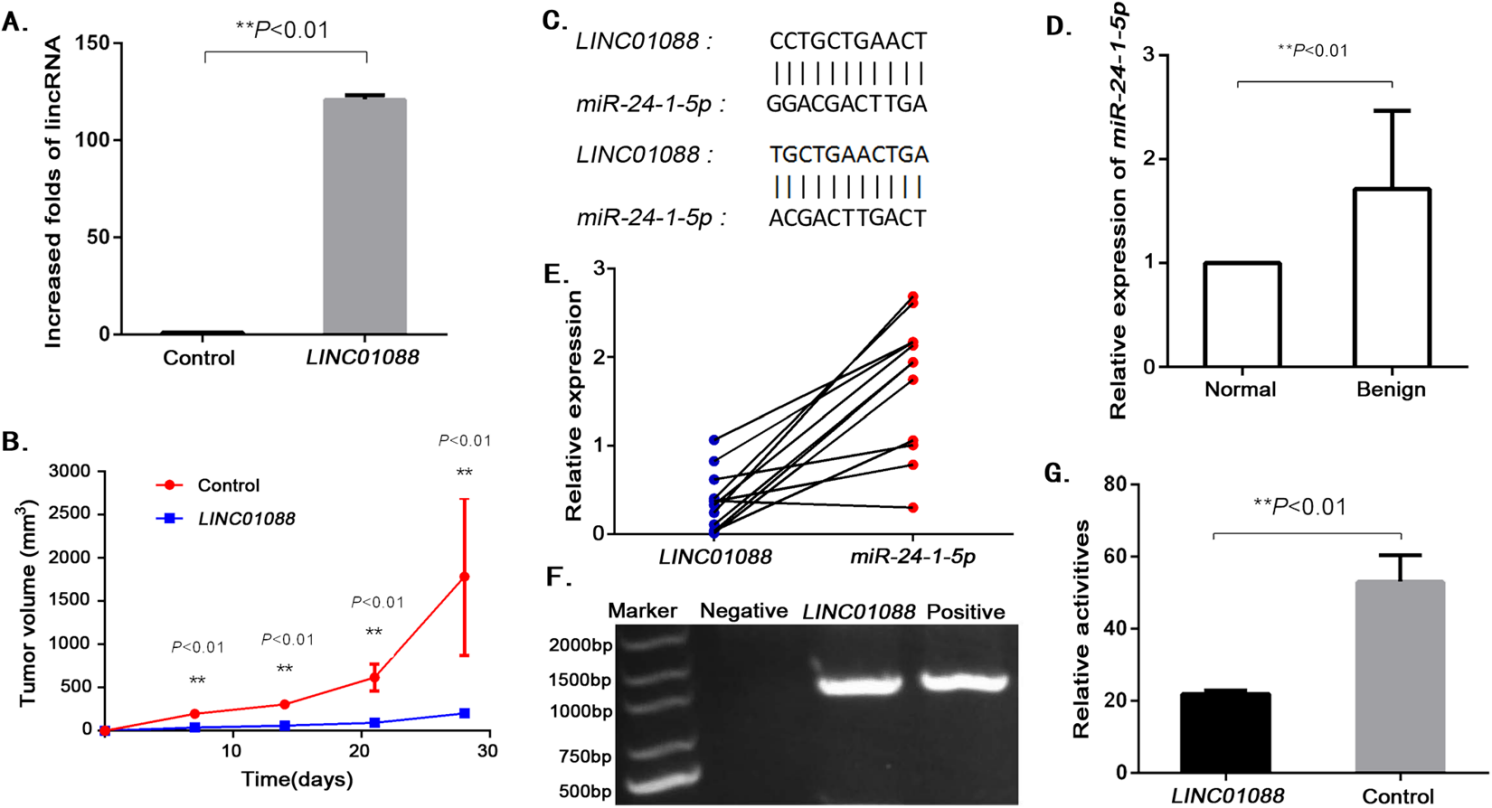
*miR-24-1-5p* is a target of *LINC01088*. (**A**) Determination of *LINC01088* in transfected cells. Total RNA was extracted from A2780 cells stably expressing *LINC01088*. Real-time qPCR was performed for detecting *LINC01088*. Data are represented as mean +/− SD, **means *P* < 0.01 vs control group (*ANOVA*). (**B**) Tumorigenicity of *LINC01088 in vivo*. Female Balb/c nude mice were subcutaneously implanted with A2780 cells transfected with *LINC01088*-lentiviral vector or blank-lentiviral vector, respectively. 28 days after implantation, the nude mice in each group were sacrificed. The tumor volume was calculated. Data are represented as mean +/−SD, **means *P* < 0.01 *vs* control (*Repeated Measure ANOVA*). (**C**) Prediction of binding sites between *LINC01088* and *miR-24-1-5p*. Gene Blasting was performed and found two potential binding sites between *LINC01088* and *miR-24-1-5p*. (**D**,**E**) Level of *miR-24-1-5p* in primary epithelial ovarian tumors. RNA was isolated from the clinical specimens using Trizol. After reverse transcription, real-time qPCR was conducted to determine the *miR-24-1-5p* level in 12 benign epithelial ovarian tumor tissue specimens and the matched normal tissues. Data are represented as mean +/− SD, **means *P* < 0.01 vs normal group (*ANOVA*) and showed a negative relation between the expression *LINC01088* and *miR-24-1-5p* (spearman rank correlation was −0.568, *P* < 0.01). (**F**) RNA co-precipitation. A2780 cells were transiently transfected with *miR-24-1-5p* expression plasmids labeled with biotin, PCR was performed to detect *LINC01088* after reverse transcription through Oligo dT15. Results showed that there was an interaction between *LINC01088* and *miR-24-1-5p*. (**G**) Validating the interaction between *LINC01088* and *miR-24-1-5p*. The relative luciferase activity was notably decreased in A2780 cells co-transfected with *pcDNA6.2-GW/EmGFP-miR-24-1-5p*, *PRL-TK* and *pMIR-LINC01088* reporter plasmids. *miR-LacZ* was taken as the control. Data are represented as mean +/−SD, **means *P* < 0.01 *vs* control (*ANOVA*).

To determine the role of *LINC01088* in tumorigenesis of ovarian epithelial cells, we constructed *LINC01088*-lentiviral vectors and transfected them into A2780 cells. RT-qPCR verified that we have establish a cell line highly expressing *LINC01088* (Fig. 2A). The tumor growth assay showed that *LINC01088* significantly inhibited tumor growth (Fig. 2B), implying that *LINC01088* was a tumor suppressor gene, although its mechanism remained unclear. Based on the role of lncRNAs as molecular “sponges”, we speculated that *LINC01088* had target miRNAs and served as a molecular “sponge”. Thus, we used the BLAST function on www.ncbi.nlm.nih.gov and found out that *miR-24-1-5p* had two binding sites for *LINC01088* (Fig. 2C). To verify the interaction between *LINC01088* and *miR-24-1-5p*, we conducted RT-qPCR analysis using a total of 22 clinical tissue samples, and found a negative correlation between the expression levels of *LINC01088* and *miR-24-1-5p* (spearman rank correlation was −0.568, *P* < 0.01) (Fig. 2D,E). Furthermore, we transfected A2780 cells with *miR-24-1-5p* expressing plasmids labeled with biotin and tested *LINC01088* by PCR using RNA sediments “pulled-down” by co-precipitation, and the results proved the existence of *LINC01088* (Fig. 2F). For further verification, *pMIR-LINC01088* luciferase reporter plasmids were constructed and transfected into A2780 cells. The luciferase activity assay showed that *miR-24-1-5p* could significantly inhibited firefly luciferase activity (Fig. 2G), indicating that *LINC01088* could target *miR-24-1-5p*. The above experiments demonstrated that *LINC01088* might suppress cell growth by targeting *miR-24-1-5p*.

### *LINC01088* reduces cell proliferation promoted by *miR-24-1-5p*

**Figure 3:**
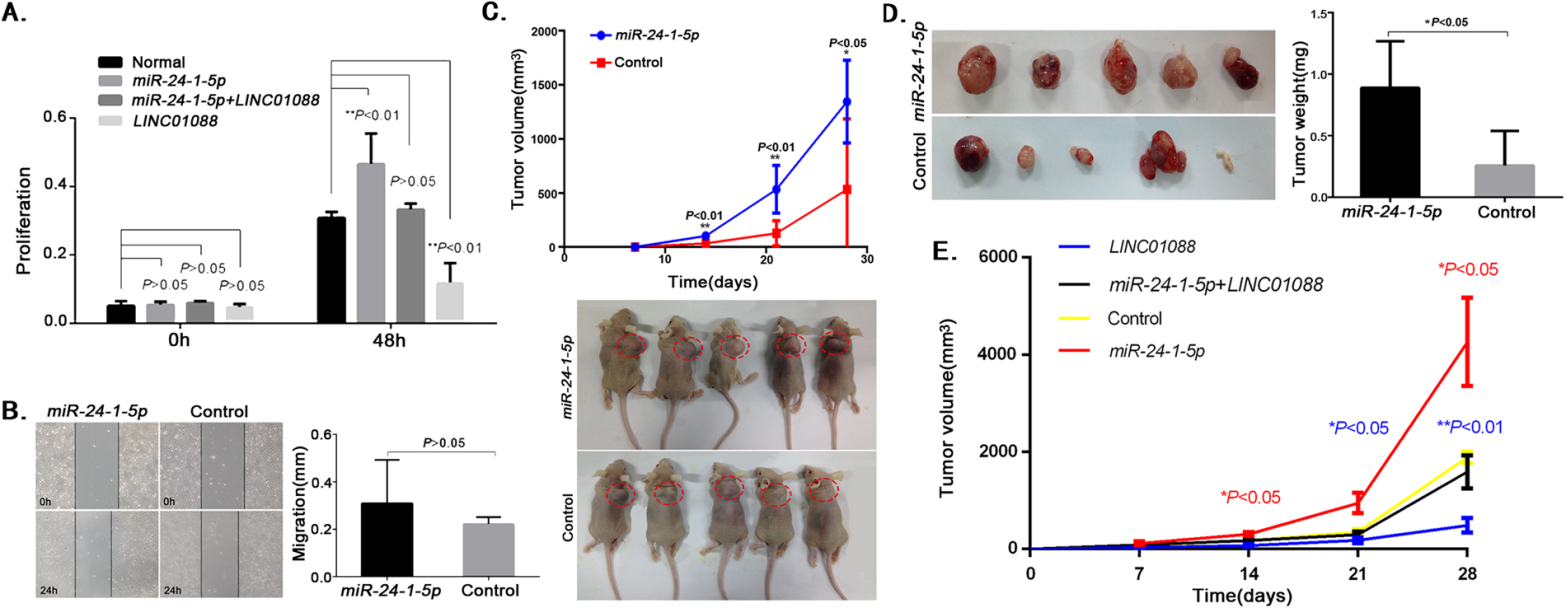
*LINC01088* reduces cell proliferation promoted by *miR-24-1-5p*. (**A**) Determination of proliferation in *LINC01088-* and *miR-24-1-5p-* expressing cells. MTS assay was performed among control group, *miR-24-1-5p* group, *miR-24-1-5p* + *LINC01088* group and *LINC01088* group to determine cell proliferation. Data are represented as mean +/− SD, ** means *P* < 0.01 vs control group (*ANOVA*). (**B**) Determination of migration in *miR-24-1-5p-*expressing cells. Scratch assay was performed to evaluated cell migration. Data are represented as mean +/− SD, *P* > 0.05 vs control group (*ANOVA*). (**C**,**D**) Tumorigenicity of *miR-24-1-5p* in mice. Female Balb/c nude mice were subcutaneously implanted with A2780 cells stably transfected with *pcDNA6.2-GW/EmGFP-miR-24-1-5p* or *pcDNA6.2-GW/EmGFP-miR-LacZ* controls, respectively. 28 days after implantation, the nude mice in each group were sacrificed. The tumor volume was calculated (**C**). Data are represented as mean +/−SD, *means *P* < 0.05, **means *P* < 0.01 *vs* control (*Repeated Measure ANOVA*). The tumor weights were calculated (**D**). Data are represented as mean +/−SD, *means *P* < 0.05 *vs* Control (*ANOVA*). (**E**) Tumorigenicity of *miR-24-1-5p* + *LINC01088* in mice. Female Balb/c nude mice were randomly divided into four groups (control, *miR-24-1-5p*, *LINC01088* and *miR-24-1-5p* + *LINC01088*). 28 days after implantation, the nude mice in each group were sacrificed. The tumor volume was calculated. Data are represented as mean +/−SD, *means *P* < 0.05, **means *P* < 0.01 *vs* control (*Repeated Measure ANOVA*).

The experiments described above demonstrated the interaction between *LINC01088* and *miR-24-1-5p*. Next, we went on to explore the effect of *miR-24-1-5p* on the growth of ovarian epithelial cells. Firstly, we assessed the impact of *miR-24-1-5p* on cell proliferation. Plasmids expressing *miR-24-1-5p* were transfected into A2780 cells to establish stable cell line expressing *miR-24-1-5p*. The MTS assay demonstrated that *miR-24-1-5p* cloud remarkably facilitated cell proliferation, while *LINC01088* distinctly inhibited cell proliferation promoted by *miR-24-1-5p* (Fig. 3A). Secondly, we investigated the role of *miR-24-1-5p* in cell migration, and the scratch assay showed that *miR-24-1-5p* had no regulatory effect on cell migration (Fig. 3B). At last, we explored the influence of *miR-24-1-5p* on tumorigenicity in animals. Balb/c nude mice were subcutaneously implanted with cells stably overexpressing *miR-24-1-5p*. Four weeks after implantation, it was evident that not only the tumor cell growth was markedly accelerated, but also the tumor volume and weight were significantly increased in the *miR-24-1-5p* group, compared with those in the control group (Fig. 3C,D). Moreover, we performed *in vivo* experiment with cells stably overexpressing *miR-24-1-5p* + *LINC01088*, the result showed that *LINC01088* inhibited tumor growth facilitated by *miR-24-1-5p* (Fig. 3E). These findings suggested that *miR-24-1-5p* promoted cell proliferation but did not affect cell migration, hinting that *miR-24-1-5p* was an oncogene and *LINC01088* could reduce cell proliferation promoted by *miR-24-1-5p*.

### *PAK4* is the potential target of *miR-24-1-5p*

**Figure 4:**
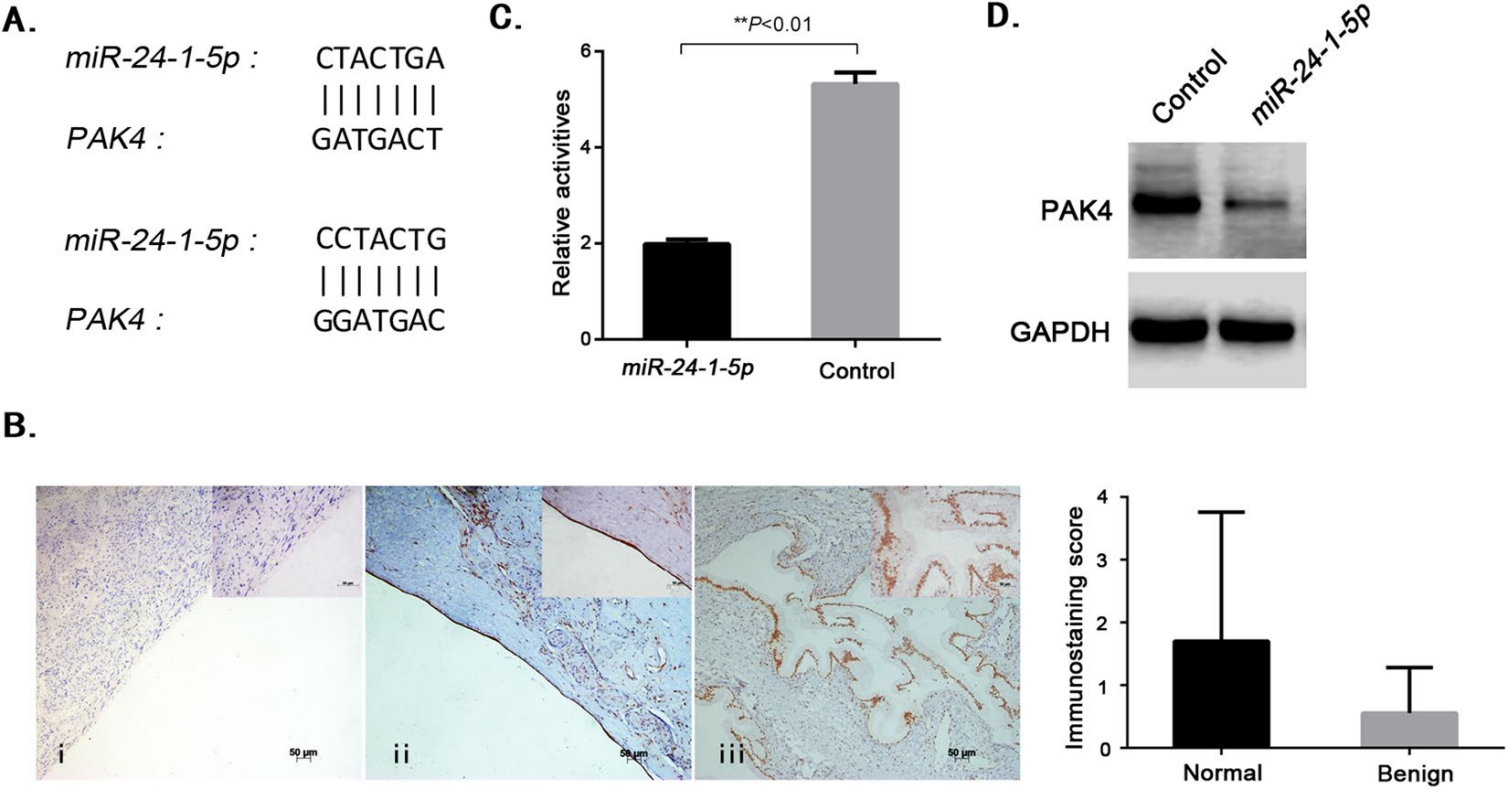
*PAK4* is the potential target of *miR-24-1-5p*. (**A**) Prediction of binding sites between *miR-24-1-5p* and *PAK4*. Gene Blasting was performed and two potential binding sites between *miR-24-1-5p* and *PAK4* were found. (**B**) PAK4 in primary epithelial ovarian tumors. IHC staining was performed to measure PAK4 expression in (ii) normal ovarian epithelial tissue, (iii) benign epithelial ovarian tumor tissue, while (i) was shown as the negative control (100X, bar = 50 um). The higher magnification (400X, bar = 50 um) was shown in the upper right corner, correspondingly. (**C**) Validating the interaction between *miR-24-1-5p* and *PAK4*. The relative luciferase activity was notably decreased in A2780 cells co-transfected with *pcDNA6.2-GW/EmGFP-miR-24-1-5p*, *PRL-TK* and *pMIR-PAK4* 3′UTR reporter plasmids. *miR-LacZ* was taken as the control. Data are represented as mean +/−SD, **means *P* < 0.01 *vs* control (*ANOVA*). (**D**) Detection of PAK4 in *miR-24-1-5p*-expressing cells. Western blot was conducted to examine the protein expression of PAK4 in A2780 cells stably transfected with *pcDNA6.2-GW/ EmGFP-miR-24-1-5p* or control plasmids (cropped; full length blots can be found in Supplementary Fig. S1).

It had previously been demonstrated that *miR-24-1-5p* increased in benign epithelial ovarian tumors and promoted cell proliferation, and was also involved in the development of epithelial ovarian tumors. Hence, we studied the target genes downstream of *miR-24-1-5p* further. Gene ‘BLAST’ on NCBI suggested that *PAK4* was the possible target of *miR-24-1-5p* (Fig. 4A). To test this assumption, firstly we performed a IHC assay on normal ovarian epithelial tissue sections and benign epithelial ovarian tumor tissue sections. The results showed that PAK4 was expressed in both normal ovarian epithelial tissues and benign epithelial ovarian tumor tissues, but PAK4 expression seemed to be lower in benign epithelial ovarian tumor (Fig. 4B). Next, *pMIR-PAK4* 3′UTR luciferase reporter plasmids were constructed and transfected into A2780 cells. The luciferase activity assay showed that *miR-24-1-5p* inhibited firefly luciferase activity dramatically (Fig. 4C). Lastly, we detected PAK4 expression in *miR-24-1-5p*-expressing cells. The western blotting data indicated that PAK4 expression in A2780 cells stably transfected with *miR-24-1-5p* recombinant plasmids was indeed lessened (Fig. 4D), while *LINC01088* had no direct effect on the expression of PAK4 (data not shown). Taken together, these findings demonstrated that *PAK4* was the target of *miR-24-1-5p*, indicating that *miR-24-1-5p* might participate in the development and progression of epithelial ovarian tumors by targeting *PAK4*.

### PAK4 inhibits cell proliferation

**Figure 5:**
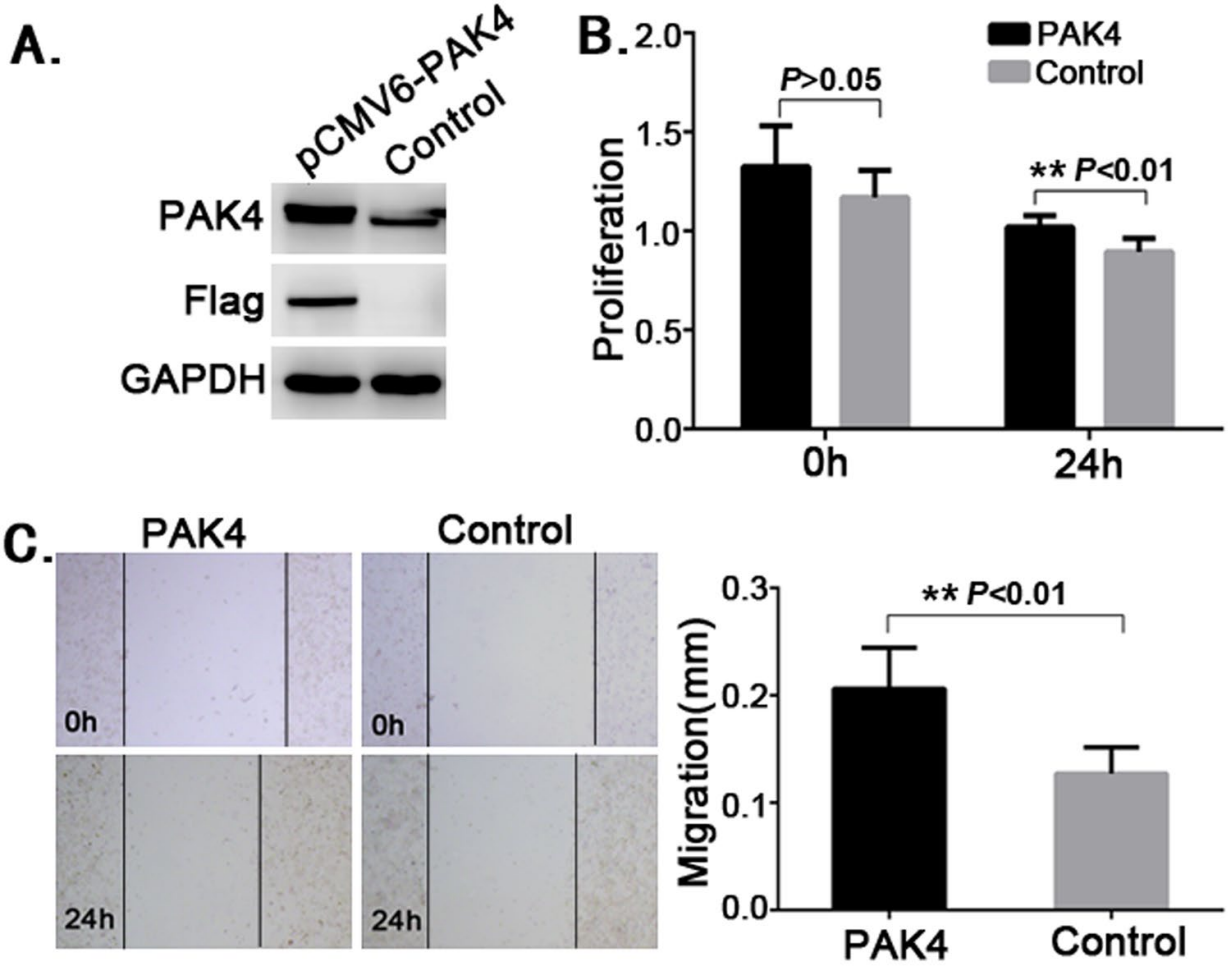
PAK4 inhibits cell proliferation. A2780 cells transfected with *pCMV6-Entry-PAK4* or control plasmids for 48 h were harvested to perform the experiments as follows. (**A**) Detection of PAK4 in stable PAK4-expressing cells. Western blot was conducted to examine the protein expression of PAK4 in two groups (cropped; full length blots can be found in Supplementary Fig. S2). (**B**) Determination of proliferation. MTS assay was performed to determine cell proliferation. Data are represented as mean +/− SD, ** means *P* < 0.01 *vs* control group (*ANOVA*). (**C**) Determination of migration. Scratch assay was performed to determine cell migration. Data are represented as mean +/− SD, ** means *P* < 0.01 *vs* control group (*ANOVA*).

To further study the biological function of PAK4 in ovarian epithelial cells, we transfected A2780 cells with plasmids expressing PAK4 (Fig. 5A), and evaluated their proliferation and migration abilities respectively. The data showed that overexpressed PAK4 significantly decreased cell proliferation (Fig. 5B), but promoted cell migration (Fig. 5C), which implied that PAK4 was a cell proliferation-inhibiting protein.

## Discussion

**Figure 6:**
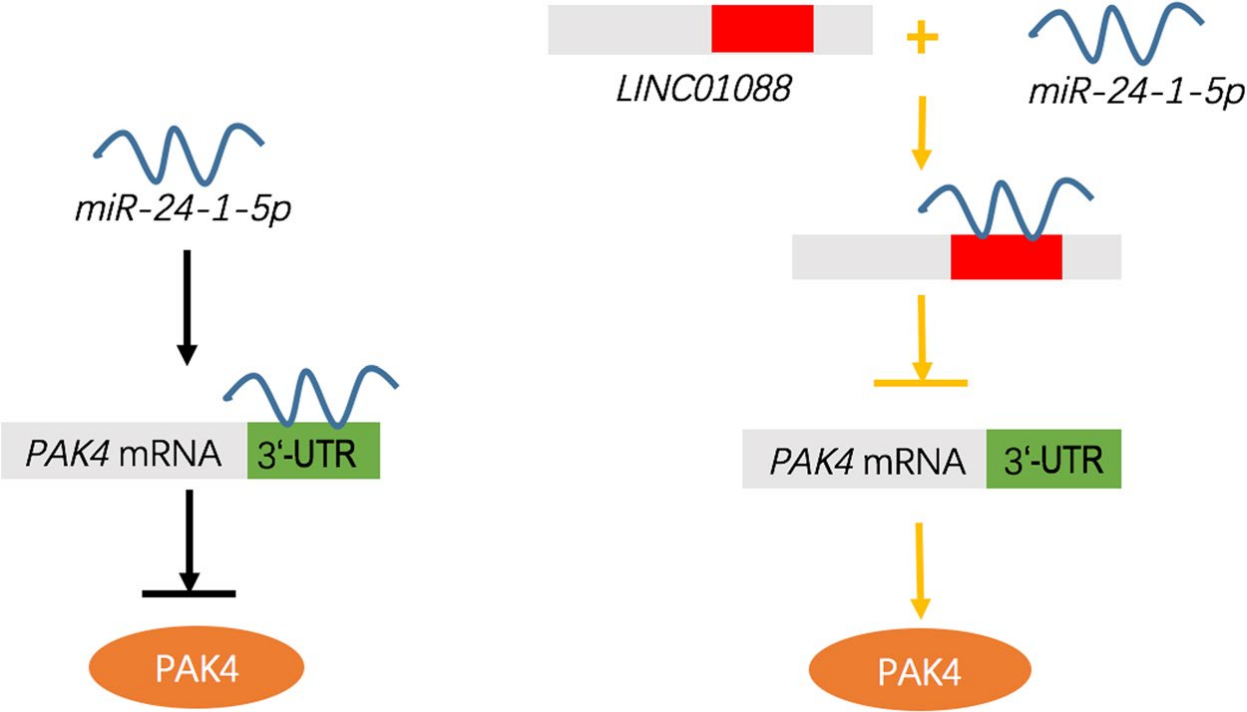
Schematic representation of the interaction among *LINC01088*, *miR-24-1-5p* and *PAK4*.

This study demonstrates that the expression of *LINC01088* is lowered in benign epithelial ovarian tumors compared to normal ovarian epithelial tissues, and *LINC01088* expression has a negative correlation with that of *miR-24-1-5p*. The study also proves that *LINC01088* can target *miR-24-1-5p* to regulate the expression of PAK4, and influence cell proliferation and migration. The brief diagrammatic representation is made in the Fig. 6. This implies that *LINC01088* might inhibit the development of epithelial ovarian tumors through its interaction with *miR-24-1-5p* and the downstream effector protein PAK4.

LncRNAs perform their biological function through regulation of gene expression. Their interaction with miRNAs is very important in tumorigenesis and tumor progression^14–17^, and lncRNA-mediated sponge regulatory network in miRNAs and pri-miRNAs is one of the modes of action. For example, Kumar *et al*.^18^ proved that *Hmga2* promoted lung cancer progression by competing for *let-7* occupancy with *TGF-beta type III receptor (Tgfbr3t)* through *transforming growth factor-β (TGF-β)* signaling pathway. Also, lncRNA*GAS5* acted as a tumor suppressor in hepatocellular carcinoma through negative regulation of *miR-21* to up-regulate its targets programmed cell death 4 (PDCD4) and phosphatase and tensin homologue (PTEN), resulting in inhibition of cancer cell migration and invasion^19^. In our study, we demonstrated that *LINC01088* targeted *miR-24-1-5p* to regulate the expression of PAK4, but the exact mechanism of the interaction between *LINC01088* and *miR-24-1-5p* required further exploration.

*MiR-24* was initially discovered through a research involving invertebrates and vertebrates by Lagos-Quintana *et al*.^20^ in 2001. *Has-mir-24* has two forms, *mir-24-1* and *mir-24-2*, located on Chromosome 9 and Chromosome 19 respectively^21^, which includes three mature sequences, *miR-24-1-5p*, *miR-24-3p* and *miR-24-2-5p*. To date, the action mechanism of *miR-24-1-5p* in carcinomas remains unclear. As reported in Braoudaki’s retrospective study in patients with ependymoma (EP)^22^, *miR-24-1-5p* was up-regulated significantly in relapse and progression cases compared to clinical remission and survival cases. Moreover, *miR-24-1-5p* was considered as an oncogene associated with multiple endocrine neoplasia type1 (MEN1)^23^. While Goto *et al*.^24^ indicated that *miR-24-1-5p* could clearly inhibit cell proliferation, migration and metastasis in prostate cancer. Inoguchi *et al*.^25^ also verified that *miR-24-1-5p* inhibited bladder cancer cell proliferation by targeting forkhead box protein M1 (FOXM1). However, the possible roles of *miR-24-1-5p* in epithelial ovarian tumors haven’t been reported yet. This study confirmed that *miR-24-1-5p* could promote epithelial ovarian tumors by negatively regulating PAK4 expression. In fact, miRNAs have innumerable target molecules, and regulate every aspect of cellular activity, such as cell proliferation, differentiation, apoptosis and so on^26–29^. Furthermore, miRNAs play multiple roles in development and progression of tumors, including angiogenesis, metastasis, and also exosome-mediated regulation^30–34^. Hence, it can be concluded that the results of our study elucidate only one of the countless signaling pathways involved in various biological processes associated with tumors, therefore further studies are required.

During the course of this study, it was demonstrated that PAK4 was the target of *miR-24-1-5p*. PAK is a class of evolutionarily conserved serine/threonine protein kinases^35^, and PAK4 is the earliest and most profoundly studied protein of the group II PAKs. The PAK4 gene is located on chromosome 19 at the 19q13 locus; it is 3064 bp in length and encodes 591 amino acids. Recent studies on PAK4 reveal its significant role in controlling cellular activities. It is considered to be a signaling integrator, regulating numerous fundamental cellular processes, such as actin cytoskeletal dynamics, cell morphology and motility, cell survival, embryonic development, immune defense and oncogenic transformation^36,37^. Besides, it also plays key roles in gallbladder carcinoma^38^, pancreatic cancer^39,40^, colorectal cancer^41^, lung cancer^42^ and so on.

Studies show that the activation and expression levels of PAK4 plays significant roles in the development and progression of tumors. It is reported that among the 100 types of cancers affecting humans, 78% have increased expression of PAK4^43^. For example, PAK4 expression is significantly increased in breast cancer and is positively correlated with tumor progression^44^. This is inconsistent with our results which suggest that cells with increased expression of *miR-24-1-5p* and decreased expression of PAK4 demonstrate enhanced proliferation. Coarfa *et al*.^45^ characterized the proteomic footprint of a panel of 8 miRNAs by using reverse phase protein arrays (RPPA) in prostate cancer; and illustrated that for at most 12% of the proteins, the expression level was determined by direct interaction between miRNAs and target mRNAs, but for the majority of them, various factors were involved. Thus, it can be summarized that the proliferation of ovarian cancer cells is regulated by numerous factors, *miR-24-1-5p* and PAK4 are only the tip of the iceberg.

## Methods

### Clinical specimens

42 fresh clinical specimens (10 normal ovarian epithelial tissue; 12 benign, 8 borderline and 12 malignant epithelial ovarian tumor tissue) were obtained in the Department of Gynecology at the Second Affiliated Hospital, College of Medicine, Zhejiang University, between 2013 and 2016. 19 formalin-fixed, paraffin-embedded tissue sections (10 normal ovarian epithelial tissue and 9 benign epithelial ovarian tumor tissue) were provided by the Department of Pathology in the same hospital between 2011 and 2015. All patients involved in this study received neither radiation therapy nor chemotherapy before surgical resection.

### Cell lines

The human ovarian cancer cell line A2780 was purchased from the Chinese Academy of Sciences (Shanghai, China). Cells were cultured in RPMI-1640 (Gibco, USA) with 10% fetal bovine serum (FBS) (Invitrogen, USA) and 100 IU/ml gentamycin at 37 °C in a humidified atmosphere with 5% CO_2_.

### The gene chip scanning

3 normal ovarian epithelial tissue and 3 benign epithelial ovarian tumor tissue were used for RNA isolation through AMBION kit (Invitrogen, USA). After passing quality inspection, the total RNA was transcribed into double stranded cDNA, synthesized into cRNA and labeled with Cyanine-3-CTP. The labeled cRNAs were hybridized onto the microarray (Agilent SurePrint G3 Human Gene Expression v3, 8*60 K, Design ID: 072363) and scanned by the Agilent Scanner G2505C (Agilent Technologies, USA). The raw data was normalized using Extraction and GeneSpring. Differentially expressed genes were identified through fold change as well as *P* values calculated using Student’s-test. The threshold set for up- and down- regulated genes was a fold change ≥ 2.0 and a *P* value ≤ 0.01. The gene chip scanning was conducted in the laboratory of the OE Biotech Company (Shanghai, China).

### Real-time qPCR assay

RNA was isolated from the clinical specimens or cells using Trizol (Invitrogen, USA) and reverse transcription was performed using RNA and primers specific for mature *miR-24-1-5p* or *LINC01088*. The reverse transcription of *LINC01088* was conducted at 42 °C for 60 min and then 95 °C for 5 min (PrimeScript 1st Strand cDNA Synthesis Kit, TaKaRa, Japan). Real-time qPCR of the reverse transcription products of *LINC01088* was performed using Premix Ex Taq (TaKaRa, Japan). The reverse transcription of *miR-24-1-5p* was conducted at 16 °C for 30 min and then 42 °C for 30 min, inactivated by incubating at 85 °C for 5 min (TaqMan, USA). The reverse transcription products of *miR-24-1-5p* were mixed with TaqMan universal PCR master mix II (ABI, USA) and the mixture was incubated at 95 °C for 10 min, followed by 40 cycles, with an extension time of 15 s each at 95 °C and 60 s each at 60 °C. The relative *LINC01088* expression was normalized to *glyceraldehyde-3-phosphate dehydrogenase* (*GAPDH)* and the relative *miR-24-1-5p* expression was normalized to *miR-484*, and analysed by the 2^−ΔΔCt^ method.

### RNA co-precipitation

A2780 cells were transiently transfected with 5ug *miR-24-1-5p* expression plasmids labeled with biotin using Lipofectamine 3000 (Invitrogen, USA) for 48 h. The cells were then lysed in RIPA buffer and centrifuged at 14 800 rpm to remove the precipitate. A total of 30 µl biotin-avidin conjugated agarose was added to the supernatant and mixed via vortexing for 2 h at 4 °C. The mixture was then centrifuged and the supernatant was carefully discarded. 500 µl chloroform was added to the precipitate for nucleic acids extraction after it was washed thrice with RIPA buffer. PCR was performed to detect *LINC01088* after reverse transcription using Oligo dT15. *miR-155* labeled with biotin was used as negative control and *LINC01088* as positive control.

### Construction of *miR-24-1-5p* expression plasmids

The genetic sequence of mature *miR-24-1-5p* (MIMAT0000079) was identified by using Genbank. The engineered pre-miRNAs were chemically synthesized according to BLOCK-iT™ Pol II miRRNAi Expression Vector Kits (Invitrogen, USA). Top primer:*5*′*-tgctgactgatatcagctcagtaggcagttttggccactgactgactgcctactgctgatatcagt-3*′, bottom primer:*5*′*-cctgactgatatcagcagtaggcagtcagtcagtggccaaaactgcctactgagctgatatcagtc-3*′. After denaturation at 95 °C for 4 min, 4 μl of 10 nM oligonucleotides were ligated into 2 μl of 5ng/μl linearized *pcDNA6.2-GW/EmGFP-miR* plasmids (Invitrogen, USA) using T4 DNA ligase. The ligation mixture was transformed into DH5α and selected by 50 μg/ml spectinomycin. Positive colonies were amplified for plasmid extraction and DNA sequencing. Then the correct plasmids were purified to remove endotoxins for subsequent use.

### Cell transfection

A2780 cells were planted in a 6-well plate with an inoculum density of 50–60%. 3.75 μl liposomes and 5 μl plasmids with 10 μl P3000 reagent were respectively added into 125 μl OPTI-MEM culture medium (Gibco, USA) according to the instructions of Lipofectamine 3000. They were mixed thoroughly and incubated at room temperature for 5 min. The solution was then added into each well of the 6-well plate and culture for 48 h.

### Establishment of stable cell line expressing *miR-24-1-5p*

A2780 cells transfected with recombinant plasmids of *miR-24-1-5p* or *miR-LacZ* for 48 h were harvested for flow cytometry sorting based on green fluorescent protein (GFP) accumulation. Then, the GFP-positive cells were cloned by limiting dilution. After colony formation, blasticidin at 7 μg/ml was used for selecting resistant cells by 14 days of culture. Blasticidin-resistant colonies were picked and expanded for use.

### Western blotting

Cells were washed with phosphate-buffered saline (PBS) and lysed in 500 μl RIPA buffer with 5 μl protease inhibitor cocktail (100X, Merck, USA) on ice for 15 min, then centrifugation at 13 000 g for 10 min. The proteins in 20 μl supernatant were separated with 12% SDS-PAGE and transferred onto a nitrocellulose membrane. Then the membrane was incubated with rabbit anti-human primary antibody PAK4 (1:500–1:1000, Abcam, UK), mouse Anti-Flag Tag (1:1000–1:10 000, Proteintech, USA) and mouse anti-human primary antibody GAPDH (1:5000, KangChen Bio-tech Inc. China) at 4 °C overnight, respectively. Next, it was incubated with a horseradish peroxidase-labeled (HRP-labeled) goat anti-rabbit or anti-mouse secondary antibody (1:2000, Jackson Immunotech, UK) at room temperature for 1 h. The membrane was developed using enhanced chemiluminescence (ECL, Millipore, Germany) for testing.

### Luciferase reporter gene assay

The intact RNA was extracted using Trizol.*LINC01088* (located in *Chr4*, size 1011 bp) were chemically synthesized. Forward primer: 5′- ctagtccccttgaaggaataggagtagacctgctgaactatcacatgagagaagaggcccaaa-3′, reverse primer: *5*′-agcttttgggcctcttctctcatgtgatagttcagcaggtctactcctattccttcaagggga-*3*′. DNA fragments of the *PAK4* 3′UTR were chemically synthesized using cDNA as a template. Forward primer: *5*′-*cagctctactagtccctcaaccaaagagccccc*-*3*′, reverse primer: *5*′-*cagtgacaagctttgtctccccatccagccaca*-*3*′. The double-stranded oligonucleotides were then ligated into the Spe I/HindIII sites in the *pMIR*-report Luciferase plasmid (Invitrogen, USA). After DNA sequencing, recombinant *pMIR-PAK4* or *pMIR-LINC01088* plasmids were co-transfected with *pcDNA6.2-GW/EmGFP-miR* plasmids containing *miR-24-1-5p* and *PRL-TK* plasmids (10:1:0.1) into A2780 cells using Lipofectmine3000 and incubated for 48 h. Then luciferase reporter gene assay was conducted by using the Dual-Luciferase Reporter Assay System (Promega, USA).

### Immunohistochemistry

Formalin-fixed, paraffin-embedded tissue sections were deparaffinized, hydrated and soaked in 3% H_2_O_2_ at room temperature for 10 min. Then the slides were incubated with rabbit anti-human PAK4 antibody (10 µg/ml, Abcam, UK) overnight at 4 °C in a humidified chamber. The negative control was obtained by omitting the primary antibody. The next day, biotin-labeled sheep anti-rabbit IgG antibody was added to sections and incubated at 37 °C for 10 to 30 min. Then, the HRP streptavidin solution was added and the slides were incubated at 37 °C for 10 to 30 min. Finally, the slides were added with DAB solution, incubated for 5–10 min and counterstained with hematoxylin for 2 min and mounted.

Two pathologists, without access to the clinical data, independently scored the tissue staining. Positive staining was indicated by the presence of brown stain. The PAK4 expression was evaluated based on the intensity of staining. The percentage of positive cells was scored as: “0” (<5%, negative), “1” (5–25%, sporadic), “2” (25–50%, focal), or “3” (>50%, diffuse). The staining intensity was scored as “0” (no staining), “1” (weakly stained), “2” (moderately stained), or “3” (strongly stained). The PAK4 immunostaining score was calculated as the percentage positive score × the staining intensity score^46^.

### MTS assay

Cells in the experimental and control groups were plated at a density of 10 000 cells per well (100 µl) in 96-well plates. After being cultured for 24 h, the cells were incubated with 20 µl MTS (Promega, USA) at 37 °Cfor 4 h. The absorbance was read at 490 nm using a microplate reader.

### Scratch assay

Cells in two groups were implanted into 3.5 cm^2^ culture plates with approximately 90% confluence. Wounds were created with a 200 µl pipette tip and washed with PBS, then serum-free medium was reintroduced at the experiment start time point of 0 hour. The diameter of the scratch was recorded under light microscopy at 24 h.

### *In vivo* tumor growth assay

Female Balb/c nude mice (5–6 weeks old) were randomly divided into groups (control, *miR-24-1-5p*, *LINC01088* and *miR-24-1-5p* + *LINC01088*), and each group contained five mice. Cells transfected with *LINC01088-*, *miR-24-1-5p*- or *LINC01088* + *miR-24-1-5p-* overexpressing plasmids were injected subcutaneously in the dorsal flank of the nude mice. The mice were observed for tumor formation by measuring the tumor major axis (a) and minor axis (b) every 7 days. The tumor volume was calculated: v = ab^2^/2. Afterwards the mice were sacrificed by cervical dislocation on the 28th day after injection, the tumors were subsequently recovered and the weight of each tumor was determined.

### Data availability

All data generated or analysed during this study are included in this published article.

### Ethical statement

We solemnly stated that all methods were performed in accordance with the relevant guidelines and regulations. All human and animal studies have been approved by the Ethics Committee of the Second Affiliated Hospital, College of Medicine, Zhejiang University. Written informed consent was obtained from each patient prior to inclusion in the study.

## Electronic supplementary material

Supplementary information
