# Supplementary material for: LINC01088 inhibits tumorigenesis of ovarian epithelial cells by targeting miR-24-1-5p
Source: Sci Rep. 2018 Feb 13;8:2876. doi: 10.1038/s41598-018-21164-9 (PMC5811426; doi:10.1038/s41598-018-21164-9)

**<Supplementary information>**

***LINC01088* inhibits tumorigenesis of ovarian epithelial cells**

**by targeting *miR-24-1-5p***

**Weijiang Zhang^1^,** **^†^, Jing Fei^1^,** **^†^, Shuqian Yu^1^, Jiayu Shen^1^, Xiaoqing Zhu^1^, Annapurna Sadhukhan^1^, Weiguo Lu^2,*^ and Jianwei Zhou^1,*^**

**^1^ Department of Gynecology, the Second Affiliated Hospital, College of Medicine, Zhejiang University, Hangzhou, Zhejiang, 310051, China**

**^2^ Department of Gynecologic Oncology, Women's Hospital, College of Medicine, Zhejiang University, Hangzhou, Zhejiang, 310006, China**

***Corresponding. Dr. Jianwei Zhou. Email:2195045@zju.edu.cn; Tel: +86-571-87783128; Fax: +86-571-87783128; Correspondence may also be addressed to Dr. Weiguo Lu. Email:lbwg@zju.edu.cn; Tel: +86-571-87031130; Fax: +86-571-87061878.**

**† These authors contributed equally to this work.**

**Supplementary Fig. S1. Full-length images of the immunoblots in Figure 4.** Black dot line boxes indicate the cropped images used in Figure 4.


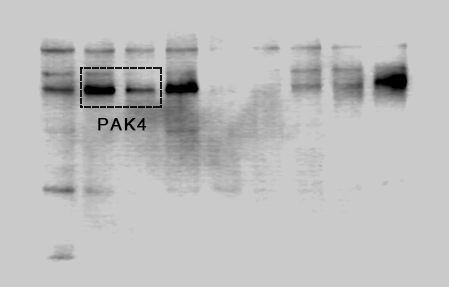


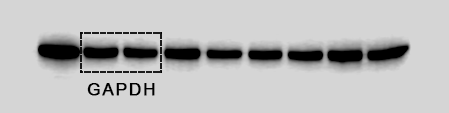


**Supplementary Fig. S2. Full-length images of the immunoblots in Figure 5.** Black dot line boxes indicate the cropped images used in Figure 5.


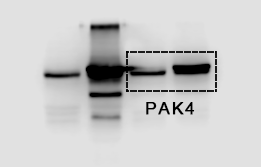


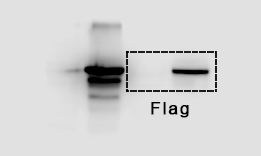


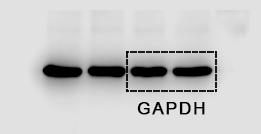

Supplement: Supplementary file 1 — Supplementary information [file 41598_2018_21164_MOESM1_ESM.doc]
